# Development of an Aquaporin-4 Orthogonal Array of Particle-Based ELISA for Neuromyelitis Optica Autoantibodies Detection

**DOI:** 10.1371/journal.pone.0143679

**Published:** 2015-11-24

**Authors:** Francesco Pisani, Paolo Settanni, Stefania Rosito, Maria Grazia Mola, Raffaele Iorio, Carla Tortorella, Maddalena Ruggieri, Maria Trojano, Maria Svelto, Antonio Frigeri, Grazia Paola Nicchia

**Affiliations:** 1 Department of Bioscience, Biotechnologies and Biopharmaceutics and Center of Excellence in Comparative Genomics, University of Bari "Aldo Moro", 70126, Bari, Italy; 2 Department of Basic Medical Sciences, Neuroscience and Sense Organs, University of Bari "Aldo Moro", 70126, Bari, Italy; 3 Department of Geriatrics, Neuroscience and Orthopedics, Institute of Neurology, Catholic University, Rome, Italy; Medical University Vienna, Center for Brain Research, AUSTRIA

## Abstract

Serological markers of Nuromyelitis Optica (NMO), an autoimmune disorder of the central nervous system, are autoantibodies targeting the astrocytic water channel aquaporin-4 (AQP4). We have previously demonstrated that the main epitopes for these autoantibodies (AQP4-IgG) are generated by the supramolecular arrangement of AQP4 tetramers into an Orthogonal Array of Particles (OAPs). Many tests have been developed to detect AQP4-IgG in patient sera but several procedural issues affect OAP assembly and consequently test sensitivity. To date, the protein based ELISA test shows the lowest sensitivity while representing a valid alternative to the more sensitive cell based assay (CBA), which, however, shows economic, technical and interpretation problems. Here we have developed a high perfomance ELISA in which native OAPs are used as the molecular target. To this aim a native size exclusion chromatography method has been developed to isolate integral, highly pure and AQP4-IgG-recognized OAPs from rat brain. These OAPs were immobilized and oriented on a plastic plate by a sandwich approach and 139 human sera were tested, including 67 sera from NMO patients. The OAP-ELISA showed a 99% specificity and a higher sensitivity (91%) compared to the CBA test. A comparative analysis revealed an end-point titer three orders of magnitude higher than the commercial ELISA and six times higher than our in-house CBA test. We show that CNS-extracted OAPs are crucial elements in order to perform an efficient AQP4-IgG test and the OAP-ELISA developed represents a valid alternative to the CBA currently used.

## Introduction

Neuromyelitis optica (NMO) is an autoimmune disorder of the central nervous system (CNS) distinct from multiple sclerosis (MS). A key serological marker of NMO is an IgG autoantibody against the astrocytic water channel aquaporin-4 (AQP4-IgG)[1,2], which is particularly abundant at the blood brain barrier (BBB) level. AQP4-IgG binding to its target leads to inflammatory lesions mediated by (BBB) breakdown and lymphocyte infiltration [1,3–5].

AQP4 is a complex plasma membrane multimeric protein expressed as two major isoforms M1 (32KDa) and M23 (30KDa) that differ in their N-terminal sequence. In the plasma membrane AQP4 assembles as heterotetramers that are able to further aggregate into a supramolecular structure known as an Orthogonal Array of Particles (OAP) [6,7]. A main determinant of OAP assembly is the M1/M23 ratio [7,8]. Although other groups have shown antibodies against intracellular AQP4 peptides [9], the main AQP4-IgG target seems to be AQP4 organized into OAPs [10,11]. In particular, AQP4-IgG binding sites are conformational and are made by OAP-specific extracelluar loop interactions [12] generated by the AQP4 tetramer assembly. Furthermore, changes in spatial position of one extracellular loop (loopA) almost completely prevent AQP4-IgG binding [13]. Thus, AQP4-IgG binding sites are highly complex and sensitive.

Treatment approaches for attack prevention in NMO and MS are different. Some immune therapies for MS seem to worsen NMO, indicating the need for early, accurate diagnosis [14,15]. The International Panel for NMO Diagnosis (IPND) has recently introduced new diagnostic criteria [16] based on the presence of AQP4-IgG in the patients’ serum. The new nomenclature unifies the terms NMO and NMO spectrum disorders (NMOSD) further divided into NMOSD with AQP4-IgG, without AQP4-IgG, and with unknown AQP4-IgG status. These new criteria, in which AQP4-IgG assumes a central role, underline the need to improve the tests for high sensitive serological AQP4-IgG detection.

To date many serological tests have been proposed which include immunofluorescence on AQP4 expressing tissues, flow cytometry, radioimmunoprecipitation assay (RIA), cell-based assay (CBA) using AQP4 expressing cells, and enzyme linked immunosorbent assay (ELISA) using recombinant AQP4 [17–24]. CBA, the most widely used test, has a sensitivity that is affected by several procedural issues. Two of them are strategic for high sensitivity: AQP4 isoform/cloning strategy and the position of a fluorescent tag [2]. Other technical issues, such as the need to use a fluorescence microscope and the use of live cells for the best results, pose a number of economic and technical problems. Consequently, it is important to explore new ways to solve these criticisms. The criticisms of CBA could be potentially solved by a protein-based NMO-IgG detection method, such as ELISA. Despite commercial and homemade ELISA having already been developed, sensitivities are too low to represent a valid alternative to CBA [24–30].

**Fig 1:**
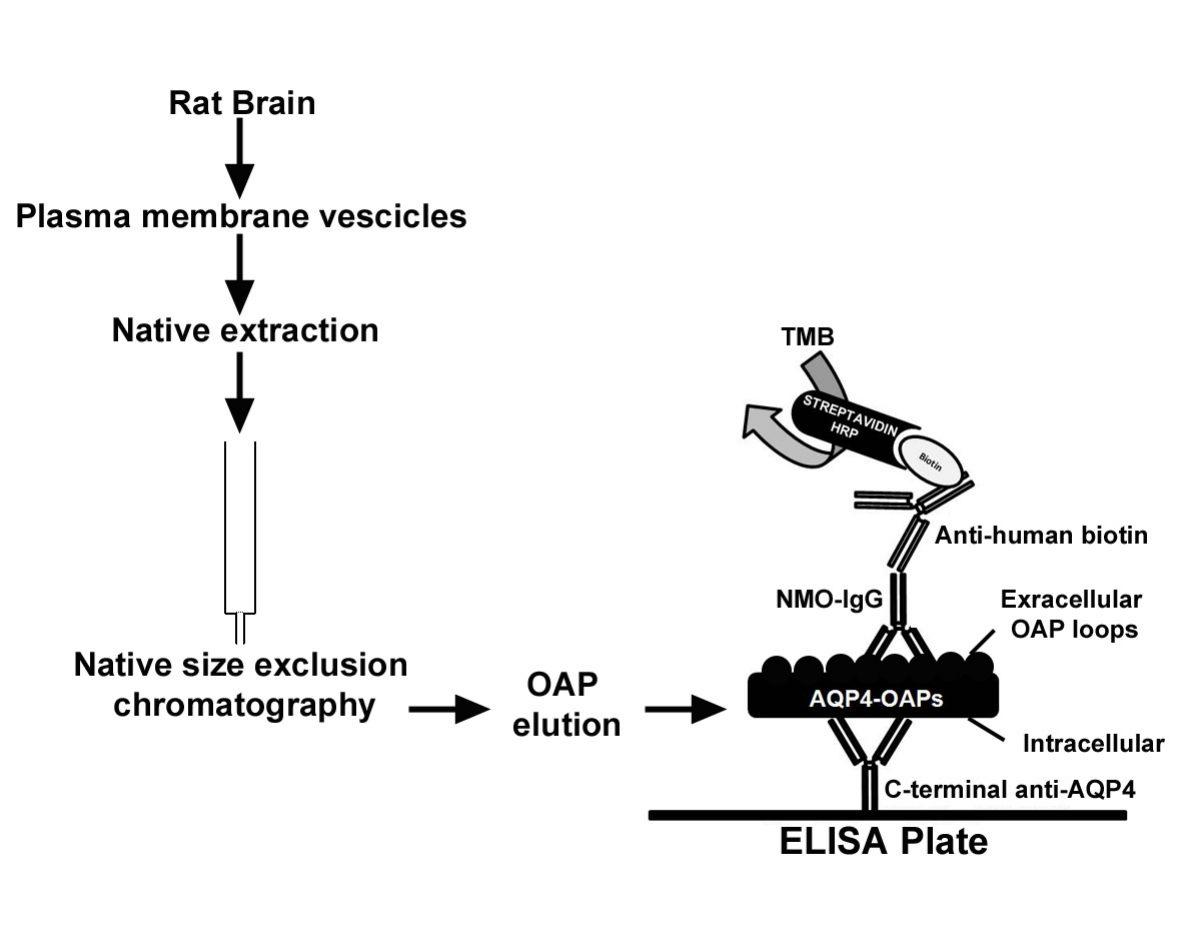
OAP-ELISA development workflow. Plasma membrane proteins were extracted under native conditions from rat brain plasma membrane vescicles and subjected to nSEC to isolate AQP4-OAPs. Native AQP4-OAPs were then immobilized on a plastic plate using a commercial AQP4 antibody with the sandwich approach. Indirect anti-human-biotin/streptavidin-HRP based AQP4-IgG detection was performed. Note that the commercial antibody recognizes the intracellular region of AQP4, while AQP4-IgG autoantibodies recognize the extracellular regions. Thus, the C-terminal anti-AQP4 antibody allows the correct orientation of AQP4-OAPs for AQP4-IgG binding.

The aim of the present work has been to develop a sensitive and reliable ELISA test able to bypass all the problems relative to AQP4 isoforms, DNA constructs and cells. The approach here presented is based on isolation of OAPs by native size exclusion chromatography (nSEC), starting from OAP expressing tissue (brain). These native OAPs were immobilized on a plastic plate and used to develop an OAP-ELISA (Fig 1) also able to detect the autoantibodies against intracellular peptides mentioned above [9].

## Methods

### Sera

139 sera were used, 67 sera from NMO patients [31] and 72 from controls. Controls included 53 patients with definite Multiple Sclerosis (MS), 5 myasthenic, 4 with a recurrent form of myelitis, 2 with polyneuropathy, 1 with myopathy, and 7 healthy donors. The diagnosis of MS was established according to the McDonald criteria [32]. Sera analyzed were mainly from two different cohorts: the Laboratory of Neurochemistry at the Department of Basic Medical Sciences, Neuroscience and Sense Organs, University of Bari, Italy and the Institute of Neurology at the Department of Geriatrics, Neuroscience and Orthopedics, Catholic University, Rome, Italy.

### Ethics statements

#### Animals

Experiments were performed in accordance with the European directive on animal use for research and the Italian law on animal care. The project has been approved by the Institutional Care and Use Committee of the University of Bari and by the Italian Health Department (Approved Project n°100/2014-B). All experiments were designed to minimize the number of animals used and their suffering. The Wistar rats used were bred in the approved facility at the University of Bari. Rats were kept under a 12 hours dark to light cycle, constant room temperature and humidity (22 ± 2°C, 75%), with food and water ad libitum, and supplied with environmental enrichment materials, such as toys and shelters.

#### Human Subjects

All subjects gave their written informed consent to the study, which was approved by the institutional ethics committee of the University of Bari.

### Plasma membrane vescicles from rat brain

Rat brains were explanted and homogenized using a Potter apparatus in 5 volumes of ice-cold Homogenizing buffer (250mM Sucrose, 10mM Tris-HCl, pH 7.5 added with a protease inhibitor cocktail (Roche Diagnostics, www.roche.com) for protein stability). The homogenate was centrifuged at 800xg for 10 minutes at 4°C and the supernatant was first centrifuged at 2,500xg for 10 minutes and then centrifuged at 17,000xg for 45 minutes at 4°C to obtain a low speed pellet enriched in AQP4-containing plasma membrane vesicles [33].

### Extraction conditions for Native Size Exclusion Chromatography

Five different extraction conditions were tested. Proteins from vesicles were extracted on ice for one hour, vortexed every 5 minutes, in 7 volumes of Extraction Buffer (500 mM aminocaproic acid, 50 mM imidazole, 2 mM Ethylenediaminetetraacetic acid (EDTA), 3%n-Dodecyl β-D-maltoside (DDM) and protease inhibitor cocktail) added with 12mM or 150mM NaCl, and with 0%, 2% or 10% glycerol, depending on the assay. The protein lysate was centrifuged at 22,000xg for 30’ at 4°C and the supernatant was used for immunoprecipitation and nSEC experiments.

### Immunoprecipitation

The protein lysate obtained as reported in the previous paragraph was subjected to immunoprecipitation experiments using NMO and MS sera or AQP4 commercial antibodies as previously reported [12].

### Native Size Exclusion Chromatography (nSEC)

For nSEC analysis, rat brain plasma membrane vescicles were extracted in nSEC-Extraction buffer (Extraction buffer added with 150mM NaCl) and the protein content was measured by BCA protein assay (Thermo, www.thermofisher.com). Lysate was than injected into AKTA-FPLC using two types of stationary phase, Sephacryl S-500 and S-300, high prep 16/60 and 26/60 (www3.gehealthcare.com). All chromatographic phases were performed at room temperature, max 0.15MPa of column pressure, and 1ml/min of flux rate. Columns were first equilibrated with 2 column volumes of nSEC-buffer-0.15% DDM (500 mM aminocaproic acid, 50 mM imidazole, 2 mM EDTA, 0.15%DDM, 150mM NaCl), and then injected with 1 or 5ml of protein lysate(10mg/ml) in 16/60 and 26/60 columns, respectively. The absorbance at 280nm was continuously monitored and fractions of 3ml for the 16/60 column, or 8 ml for the 26/60 column were collected. Total protein content of nSEC fractions was quantified using a Micro-BCA Protein Assay Kit (Thermo, www.thermofisher.com).

### Electrophoresis, BN-PAGE, 2DE (BN/SDS-PAGE) and Western blot analysis

Thirty μl of each nSEC fraction were analyzed by immuno blot after BN-PAGE,SDS-PAGE and 2DE (BN-SDS/PAGE) as previously reported (34). Briefly, after electrophoresis proteins were blotted on PVDF membrane and revealed with anti-AQP4 antibodies (Santa Cruz Biotechnology, www.scbt.com). The secondary antibody utilized for immunoblotting analysis was a peroxidase-conjugated donkey anti-goat IgG (Santa Cruz Biotechnology, www.scbt.com).

### Dot-blot

AQP4 elution profile after nSEC was evaluated by dot blot. Two μl of each fraction were spotted on to a nitrocellulose membrane (Protran, www.sigmaaldrich.com), blocked with 5% milk in 1% Triton X-100in PBS and processed as reported for regular AQP4 imunoblotting.

### Densitometric Analysis

Densitometric analysis was performed using ImageJ software. Optical density value was determined for equal sized boxes drawn around antibody-stained bands.

### Recombinant AQP4 expression and purification

Human AQP4 with C-terminal His tag was expressed using the Bac-to-Bac expression system (Life Technologies, www.thermofisher.com) in *Spodoptera frugiperda* cells according to the instruction manual. His tagged AQP4 was purified by Nickel based chromatography using Agarose-Ni2+ NTA beads (QIAGEN, www.qiagen.com) according to the instruction manual by batch purification. Purified AQP4 was checked by SDS-PAGE and Coomassie staining, quantified by Micro-BCA Protein Assay Kit (Thermo, www.thermofisher.com) and used for quantitative immunoblot.

### Quantification of AQP4 enrichment and AQP4 purification grade in nSECfractions

Quantification of AQP4 enrichment and AQP4 purification grade in nSEC fractions were estimated by SDS-PAGE and quantitative immunoblotting, using purified AQP4 protein as standard.

In the same gel 2 and 10 ul of nSEC fractions and serial dilutions of purified AQP4 (range 0.1–10 ng) were loaded to obtain a standard curve for the calculation of AQP4 content in each nSEC fraction, based on densitometry analysis of the bands obtained. The enrichment was calculated as ng of AQP4 per ul of nSEC fraction, and purification grade as ng of AQP4 per ng of total protein (%) in each fraction.

### Commercial ELISA and OAP-ELISA

Commercial M23-based ELISA-RSR^TM^ AQP4 Ab Version2 (RSR, AQP4/96/2) was used according to the instruction manual.

For OAPs-ELISA, PBS containing 1.5% BSA (indicated as solution “A”) and agitation with 400rpm (indicated with “shaking”) were used. The OAP-ELISA here developed was perfomed as follows. Maxisorp NUNC Plates (Thermo, www.thermofisher.com) were coated with 0.2 ug of commercial goat anti-AQP4 antibody (Santa Cruz Biotechnology, www.scbt.com) overnight at 4°C or for 2 h at 37°C. After coating, wells were washed twice with 0.1% Tween-20 in PBS. Positive wells were coated with about 35ng of AQP4 diluted in 100 μl of nSEC-buffer, or after a 10-fold concentration step with Amicon 100K (Millipore). Negative control wells were only coated with 100ul of nSEC-buffer, at 4°C. After incubation, wells were saturated with solution A, for 1h. Sera were initially diluted from 1:500 to 1:1,000 in A and incubated in the wells (100ul/well) for 1h under shaking. Wells were then washed three times with A and incubated with anti-human biotynilated secondary antibody (Millipore AP112B, 1:7,000 in A) for 1h under shaking. After washings (3 x 5’ with A), streptavidin-HRP (Millipore SA202, 1:1,000 in A) was added to the wells for 1h. After five washings for 10’ with 0.1% Tween-20 in PBS, under 700rpm shaking,100ul of TMB solution was added (Millipore, www.merckmillipore.com) and incubated for 20’. The reaction was stopped by adding 100ul of 0.3M sulphuric acid solution. Finally, absorbance was read at 450nm by microplate reader. Normalized absorbance was calculated as follows: absorbance of AQP4-coated well minus absorbance of negative control well. Notably, this step was necessary, because a serum-specific empty well reactivity emerged. Absorbance was read using a FlexStation3 (Molecular Devices). The ELISA cut-off was calculated in the control group as the mean of normalized absorbance at 450nm plus three times the standard deviation.

### Sandwich OAPs-ELISA with commercial antibodies

When necessary, to detect the amount of AQP4 coating the wells, OAPs-ELISA was performed using 0.2ug/well of commercial rabbit anti-AQP4 (Santa Cruz, Santa Cruz Biotechnology, www.scbt.com) in the first coating step. Goat anti-AQP4 antibody (Santa Cruz, Santa Cruz Biotechnology, www.scbt.com) and the anti-Goat Biotin (Millipore, www.merckmillipore.com) were used in the same conditions reported in previuos paragraph.

### Cell based assay

AQP4 IgG in the sera were detected by live cell based assay using an AQP4-M23 stable trasfected cell line and an untrasfected cell line as negative control as previously reported [2]. Some sera were also tested using commercial CBA (Euroimmune). All sera were independently blind tested by two independent investigators that agreed with each other.

### Statistical analysis

Statistical analysis (means, standard deviations), significance of group differences were evaluated using GraphPad Prism 5 (GraphPad, San Diego, USA) by the student’s t-test. A p value < 0.05 was considered statistically significant. Sensitivity as a function of the specificity receiver operating characteristic (ROC) curve was generated for ELISA results. The AIC, R-Square and C-index (area under the curve) were calculated.

## Results

### Testing OAP integrity and AQP4-IgG binding in nSEC compatible buffer

**Fig 2:**
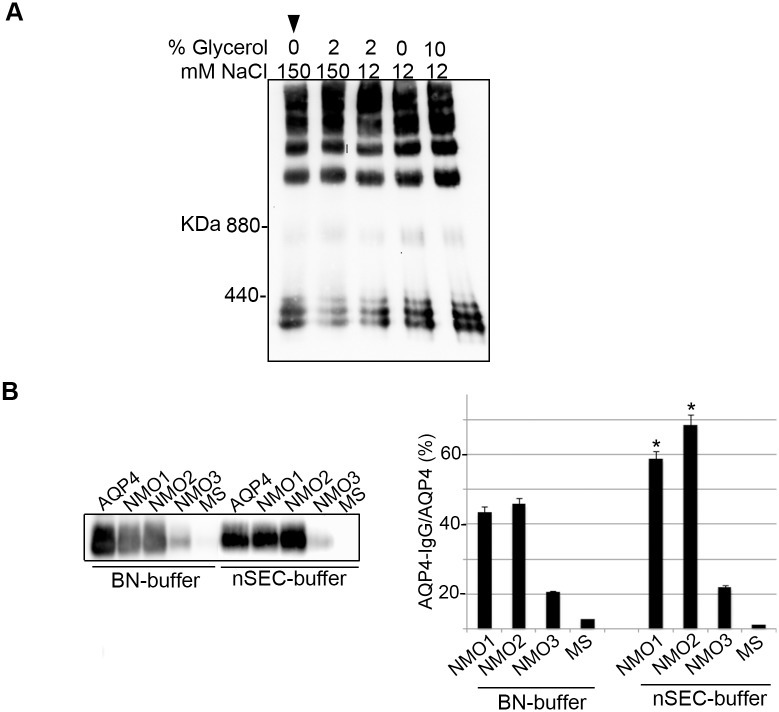
Extraction evaluation for maintaining OAP integrity and AQP4-IgG binding. (A) AQP4 immunoblotting after BN-PAGE of rat brain membrane vescicles extracted in native conditions using five different 3% DDM containing buffers. Buffers containing 0%, 2% or 10% of glycerol in combination with 12 or 150 mM NaCl were tested. Note that all tested extraction conditions were able to preserve OAP structure. (B) Left, AQP4 immunoblotting after immunoprecipitation with commercial AQP4 antibody, three NMO and one MS sera, using BN-Bufferand nSEC compatible buffer (arrowhead in A). High AQP4-IgG titer (NMO1 and 2), and low titer (NMO3) were used. Right, densitometric analysis showing the commercial anti-AQP4 normalized AQP4 immunoprecipitated by AQP4-IgG (n = 3, *p<0.05, nSEC *VS* BN-buffer).

We have previously demonstrated that the blue-native (BN) buffer is able to preserve OAP integrity and AQP4-IgG binding (11). However, BN buffer contains high glycerol and low NaCl concentration, which are incompatible with low viscosity and high ionic strength, necessary to perform an efficient nSEC. Here an nSEC compatible buffer was formulated and tested for OAP integrity and AQP4-IgG binding. Rat brain plasma membrane vescicles were extracted under native condition with BN-buffer, nSEC compatible buffer and other buffers containing intermediate conditions of viscosity and high strength, and analyzed by BN-PAGE. While the detergent concentration (3%) was maintained constant, glycerol and NaCl concentrations were modified. Fig 2A shows that all buffers tested were able to preserve OAP integrity, indicating that OAPs are highly stable structures. Then, it was tested whether the low viscosity and high ionic strength of nSEC compatible buffer would affect AQP4-IgG binding. Therefore, rat brain plasma membrane vescicles were extracted with the nSEC-buffer and with BN-buffer and AQP4-IgG binding tested by immunoprecipitation using three NMO (two high titer and one low titer) which represent three major epitopes categories [12] and MS sera (Fig 2B). Fig 2B shows that AQP4-IgG binding was higher in the nSEC-buffer compared with those observed with BN-buffer. These data demonstrate that the low glycerol and the high NaCl concentration are completely compatible with OAP intergity and AQP4-IgG binding.

### Fractionation of OAPs by nSEC using Sephacryl stationary phase

**Fig 3:**
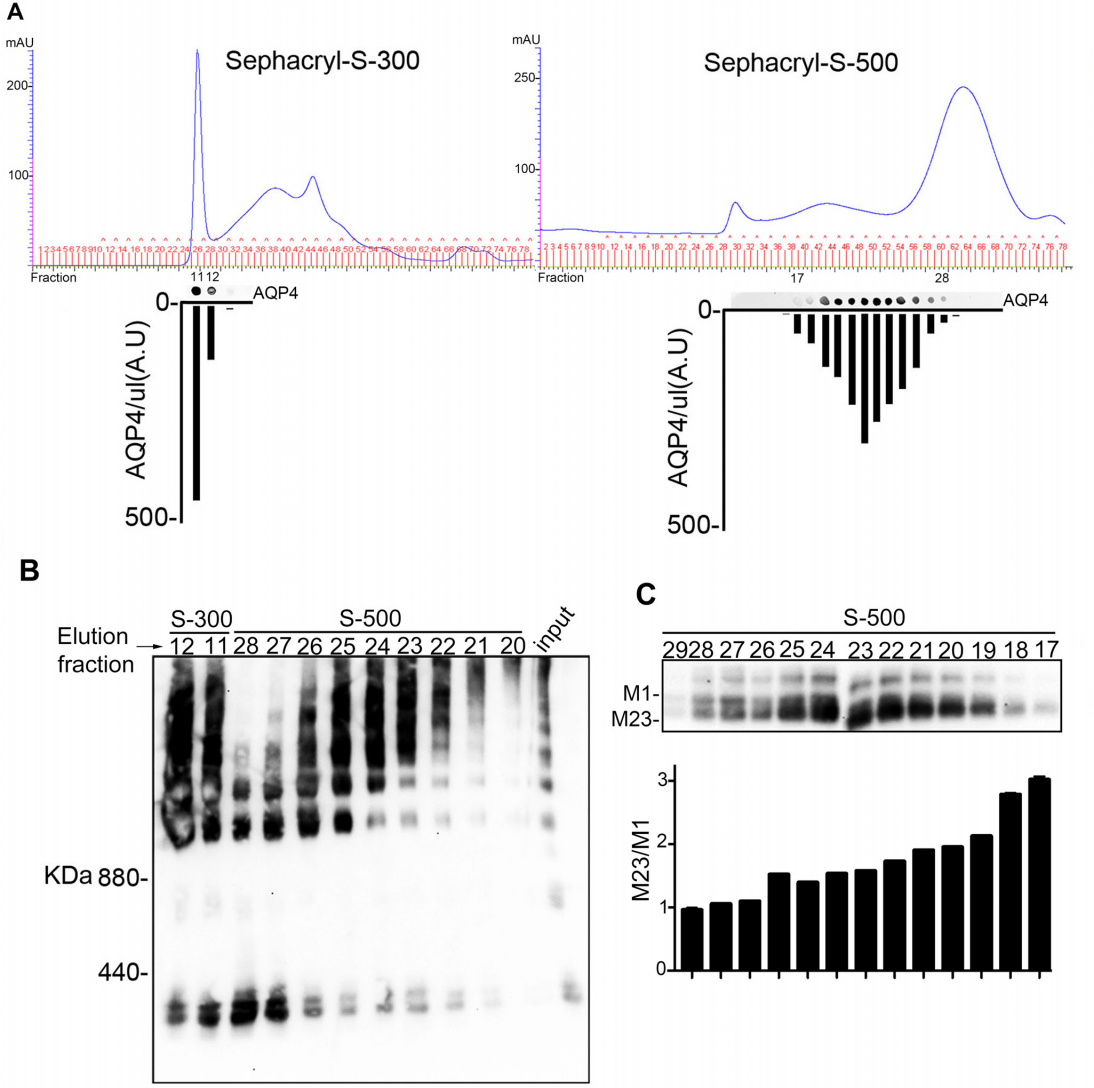
OAPpurification by nSEC. (A) Chromatograms obtained by S-300 (left) and S-500 (right)-based nSEC (columns 16/60). AQP4 levels in each fraction were evaluated by dot blot and data are reported on the graph displayed at the bottom of the chromatogram. Note that using S-300, AQP4 was only eluted in the first fractions (38-40ml), while using S-500, AQP4 was detected between 50 and 90ml. (B) BN-PAGE analysis of AQP4-OAP distribution in nSEC fractions. (C) SDS-PAGE analysis of the M23/M1 ratio in S-500 fractions. Note that the M23/M1 ratio reported in the histogram correlates with the OAP dimension of each fraction shown in B.

Plasma membrane vesicle lysate was injected into an AKTA-FPLC system and nSEC was performed using Sephacryl S-300 and S-500 columns. Fig 3A shows the elution profile of total proteins and AQP4. First peaks (excluded fraction) of both columns were contaminated by nucleic acid (data not shown). Analysis of AQP4 by dot blot showed that after fractionation using S-300, AQP4 was exclusively eluted in the first peak (38-40ml), while the S-500 column allowed a better separation between AQP4 and nucleic acids and showed several AQP4 positive fractions from 50 to 90ml. To explore OAP integrity after nSEC, AQP4 positive fractions were analyzed by BN-PAGE. Fig 3B shows that both columns were able to preserve OAP integrity, but only S-500 allowed the chromatographic fractionation of OAPs depending on supramolecular assembly sizes. As expected [34], the OAP elution profile was correlated to the M1/M23 ratio (Fig 3C). The S-500 elution profile was then used to develop the OAP-based ELISA.

### Analysis of OAP complexity and purification grade

**Fig 4:**
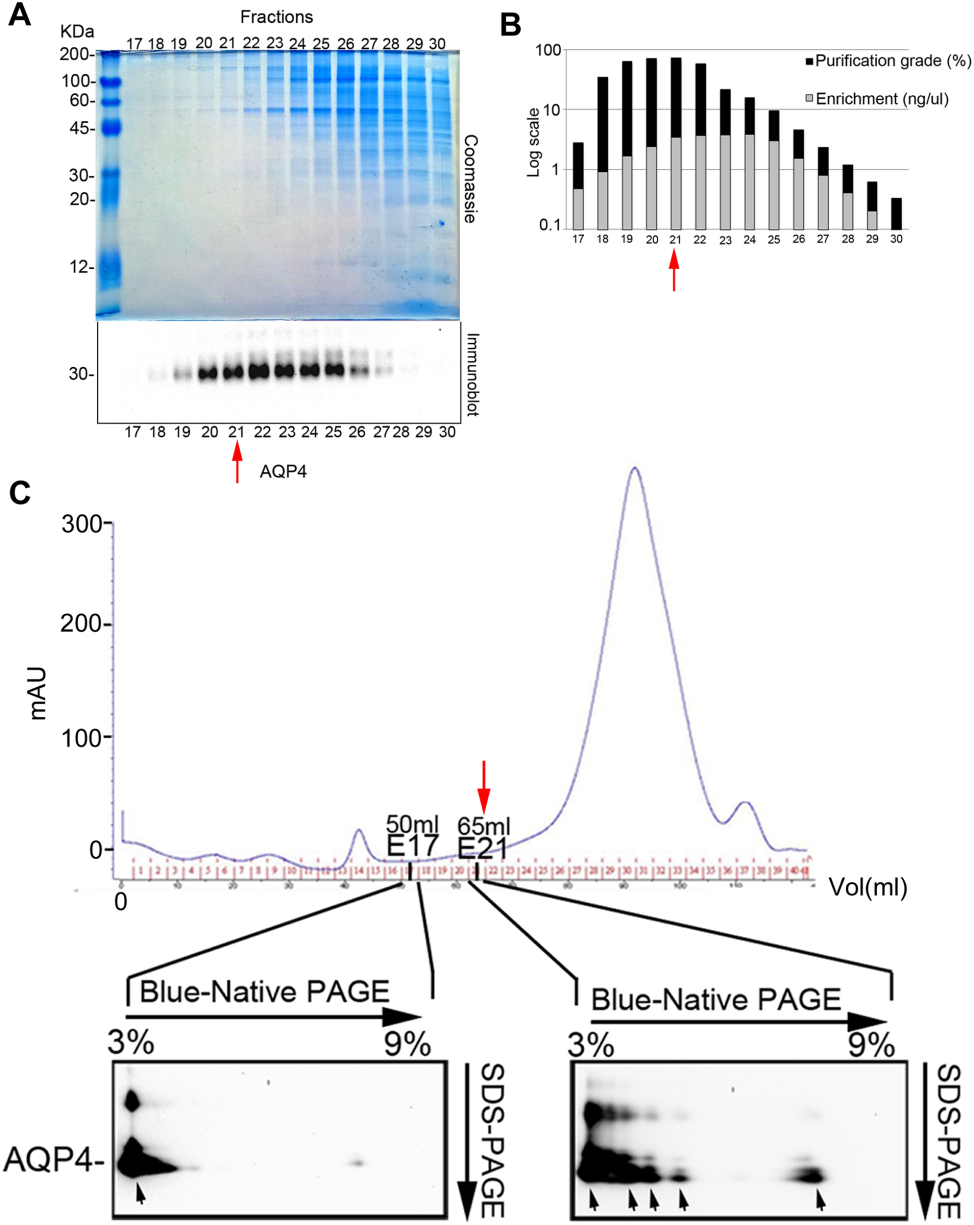
Analysis of OAP complexity and purification grade using 16/60 Sephacryl S-500-based nSEC. (A) Analysis of nSEC fractions obtained using 16/60 Sephacryl S-500. The E17-E30 fraction analysis was performed by SDS-PAGE followed by Coomassie blue staining and AQP4 immunoblotting. Note that 10 times more proteins were used for Coomassie blue staining than for AQP4 immunoblotting. (B) Quantification of AQP4 enrichment (ng of AQP4/ul) and purification grade (AQP4/total protein (%)) in fractions E17-E30. The red arrow indicates the fraction with the highest AQP4 purification and enrichment grade. (C) Chromatogram obtained by S-500 based nSEC and analysis. Fractions 17 and 21 were analyzed by 2DE and immunoblot (bottom). The red arrow indicates the position, in the SEC elution profile, of the OAP-containing fraction with maximal enrichment and purification grade used for 2DE.

The nSEC fractions were then analyzed by quantitative immunoblotting, and 2DE to identify an enriched fraction with a high molecular weight and high purification grade OAPs as well as being functionally useful for AQP4-IgG binding. Fractions in the E17-30 range (50–65 ml) were analyzed by SDS-PAGE followed by Coomassie staining and AQP4 immunodetection (Fig 4A). Between E17 and E21 a few faint protein bands were stained with Coomassie blue, while many more proteins of progressively more intensely reduced molecular weight were visible up to E30. AQP4 immunoblotting showed that E20-25 were the most AQP4-positive fractions. Quantitative analysis by immunoblotting using purified AQP4 as internal standard was performed to quantify AQP4 enrichment and purification. AQP4 enrichment was maximal between E21-E25, while AQP4 purification was maximal for fractions E20 and E21 (Fig 4B). To evaluate the size of AQP4-OAPs in two useful fractions, a 2DE analysis was performed (Fig 4C) with fractions E17 and E21. E17 (50ml) showed only one very large OAP pool, while at least five different OAP pools were identified in E21 (65 ml, arrows). In particular E21 showed a profile of OAPs similar to that found in CNS [35,36], therefore representing a good candidate for OAPs-ELISA.

### Setting up an AQP4-IgG specific OAP-ELISA sandwich

**Fig 5:**
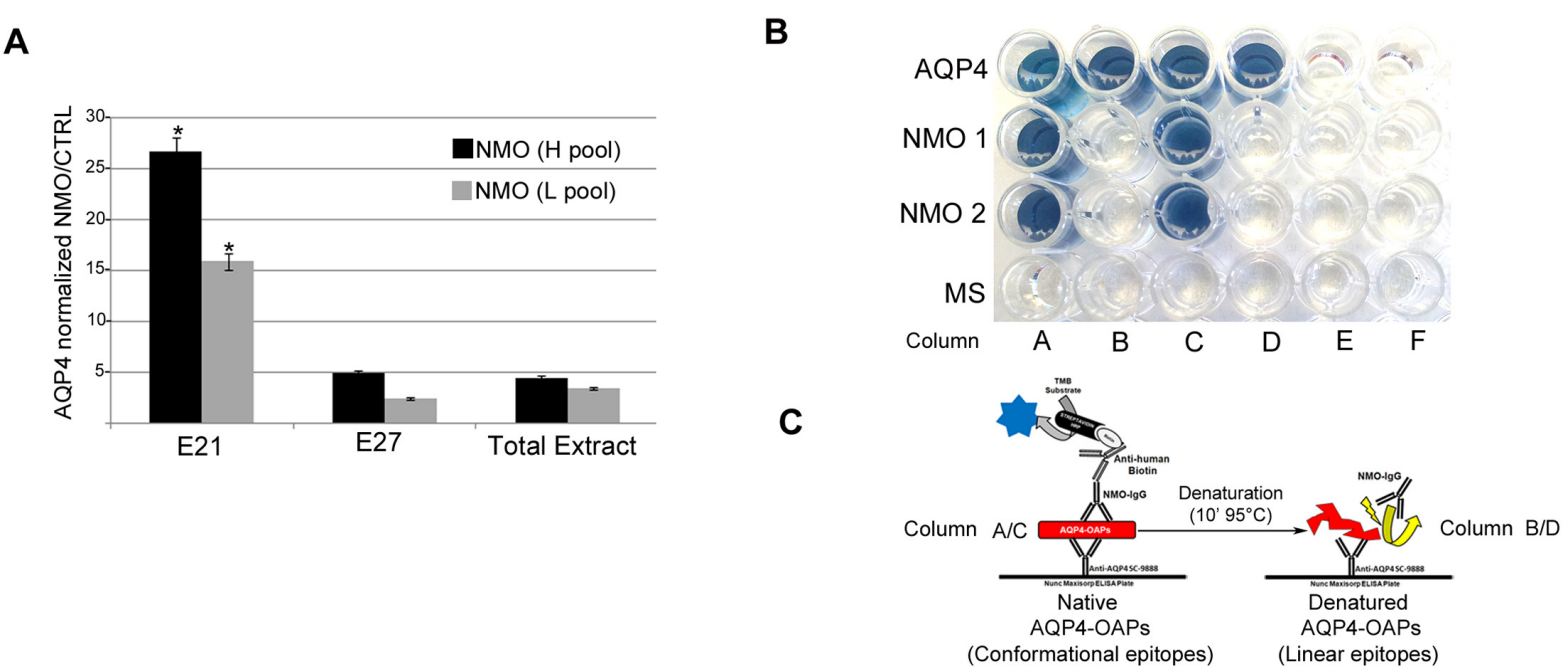
AQP4-IgG specific OAP-ELISA. (A) Analysis of AQP4 IgG binding on E21 (M23 enriched fraction), E27 (M1 enriched fraction) and total extract using a pool of High (H) and of Low (L) titer sera each from five different NMO patients. The results have been normalized on a control pool serum from five different MS patients. *P<0.01 versus E27 and total extract. (B) Representative OAP-ELISA result. Commercial Anti-AQP4 antibody-based ELISA (AQP4), two sera from AQP4 IgG positive NMO patients (NMO1 and NMO 2) and one MS control serum were analyzed using native (columns A,C) and denatured (B,D) AQP4 positive (A,B,C,D) and AQP4 negative (E) nSEC fractions (AQP4 negative fraction was E35). Empty wells were in column F. Note that AQP4-IgG binding (blue reaction in NMO 1 and NMO 2) was only obtained using AQP4 IgG positive NMO sera on native AQP4 positive fractions, while all other conditions were negative. (C) Cartoon of conformational epitope disassembly after denaturation using in-house OAP-ELISA.

We have tested whether the M23/M1 ratio and AQP4-purification grade of nSEC fraction affect specificity and selectivity of AQP4-IgG binding in ELISA sandwich. Therefore, 35ng/well of proteins from M23 enriched fraction (E21), M1 enriched fraction (E27) and membrane vesicle total extract were immobilized on an ELISA plate using a sandwich approach with a C-terminal specific anti-AQP4 antibody. High titer, low titer NMO and control sera were used, with each category composed by a pool of five different sera. The results show that the reactivity of E21 was significantly higher compared to E27 and total extract (Fig 5A), suggesting E21 as the best candidate to develop OAPs-ELISA and supporting the need for the purification technique. To investigate whether AQP4-IgG binding occurred only using native OAPs, E21 was spotted under native and denatured conditions. An anti-AQP4 commercial antibody based ELISA was performed to demonstrate the presence of AQP4 in the well. The results (Fig 5B and 5C) showed that AQP4-IgG is only able to bind native OAPs while no binding occurred using the denatured form, in agreement with the conformational nature of AQP4-IgG epitopes [2] and suggesting that the presence of auto-antibodies that react with linear peptides, identified by another group [9], could represent a minor part.

### Testing OAPs-ELISA sensitivity and specificity

**Fig 6:**
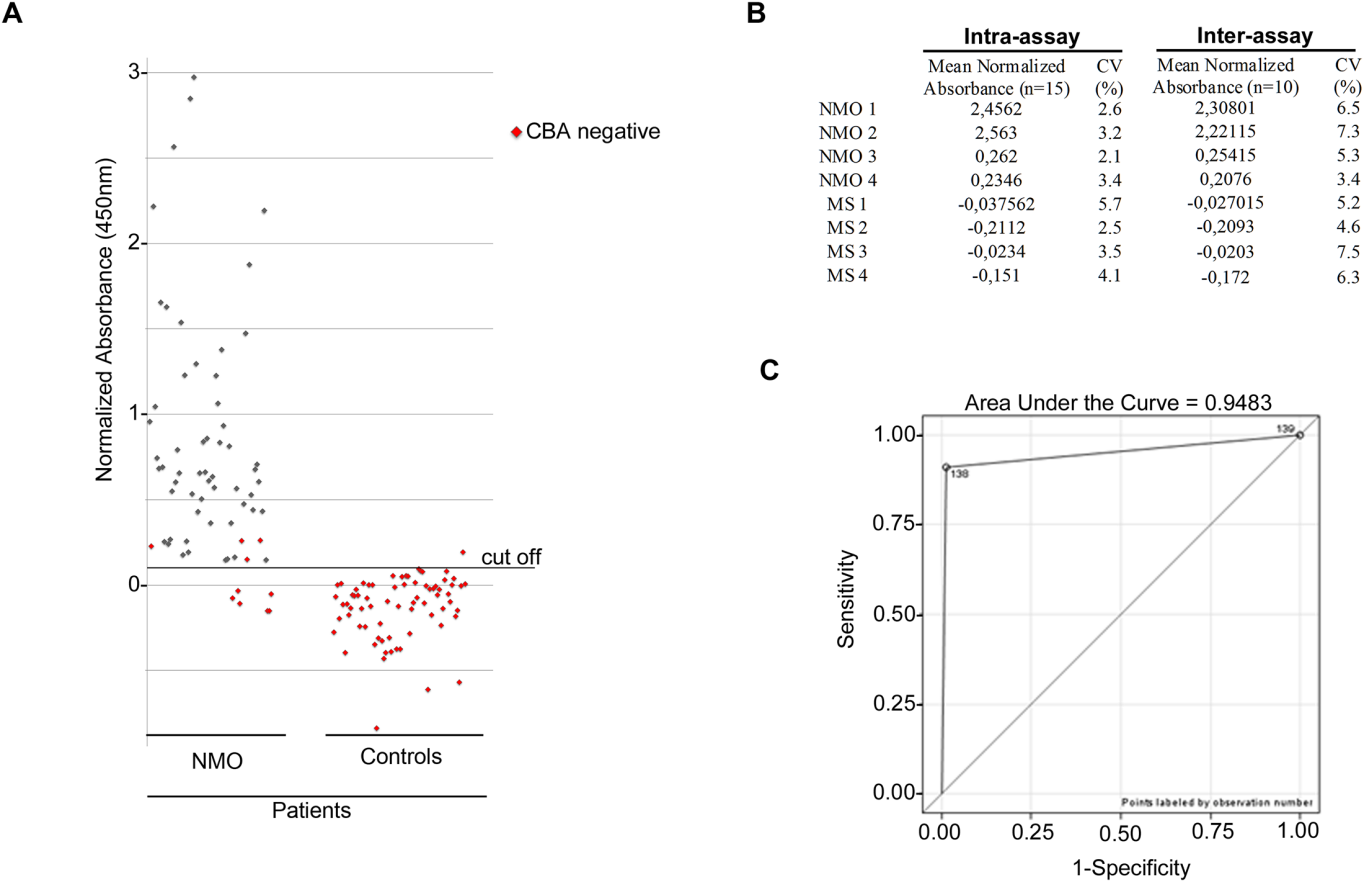
OAPs-ELISA performance. (A) Scatter plot of sera tested by OAP-ELISA. Among the 67 NMO sera analyzed, 61 were found to be positive by OAP-ELISA, while in the control group 71/72 were negative by OAPs-ELISA. The cut-off lane represents the limit value for positivity/negativity. In house CBA negative were reported in red. (B) intra- and iter-assay reproducibility of OAPs-ELISA using four NMO and four control MS sera. (C) Sensitivity as a function of specificity ROC analysis curve.

139 sera, 67 from NMO patients and 72 controls, were blind tested using our in-house CBA and OAP-ELISA (Fig 6). Using OAPs-ELISA (cut-off = 0.1), 61 NMO sera were found to be positive (61/67, 91% sensitivity), and all controls except one were negative (71/72, 99% specificity). By in—house CBA 57 NMO sera were positive (57/67, 85% sensitivity) and all controls were negative (72/72, 100% specificity). OAPs-ELISA sensitivity was also compared to commercial CBA. To this purpose, 45 of the 139 sera, 25 from NMO and 20 from controls were further retested in blind fashion with OAPs-ELISA and commercial CBA. Results showed 92% sensitivity with OAPs-ELISA and 76% with commercial CBA (S1 Table).

Importantly, the higher end-point titer of the OAP-ELISA test allowed the detection of AQP4 IgG in four sera that were negative with both in-house and commercial CBA assay. Inter- and intra-assay reproducibility of OAPs-ELISA were tested using 8 sera, 4 from NMO (two of them very close to the cut-off) and 4 from controls, showing high reproducibility (Fig 6B). Furthermore the reproducibility of the assay was also tested for four sera very close to the cut-off in both positive and negative sera (S1 Fig).

To evaluate the accuracy of the OAPs-ELISA test, a ROC analysis was performed. ROC analysis showed a large area under the curve (0.9483) supporting the high statistical efficiency of OAPs-ELISA (Fig 6C). Importantly, the OAPs-ELISA performance was substantially invariable using different preparation and frozen-thawed E21. Taken together, these data demonstrate the high reliability of OAPs-ELISA.

### End-point titer of OAPs-ELISA

**Fig 7:**
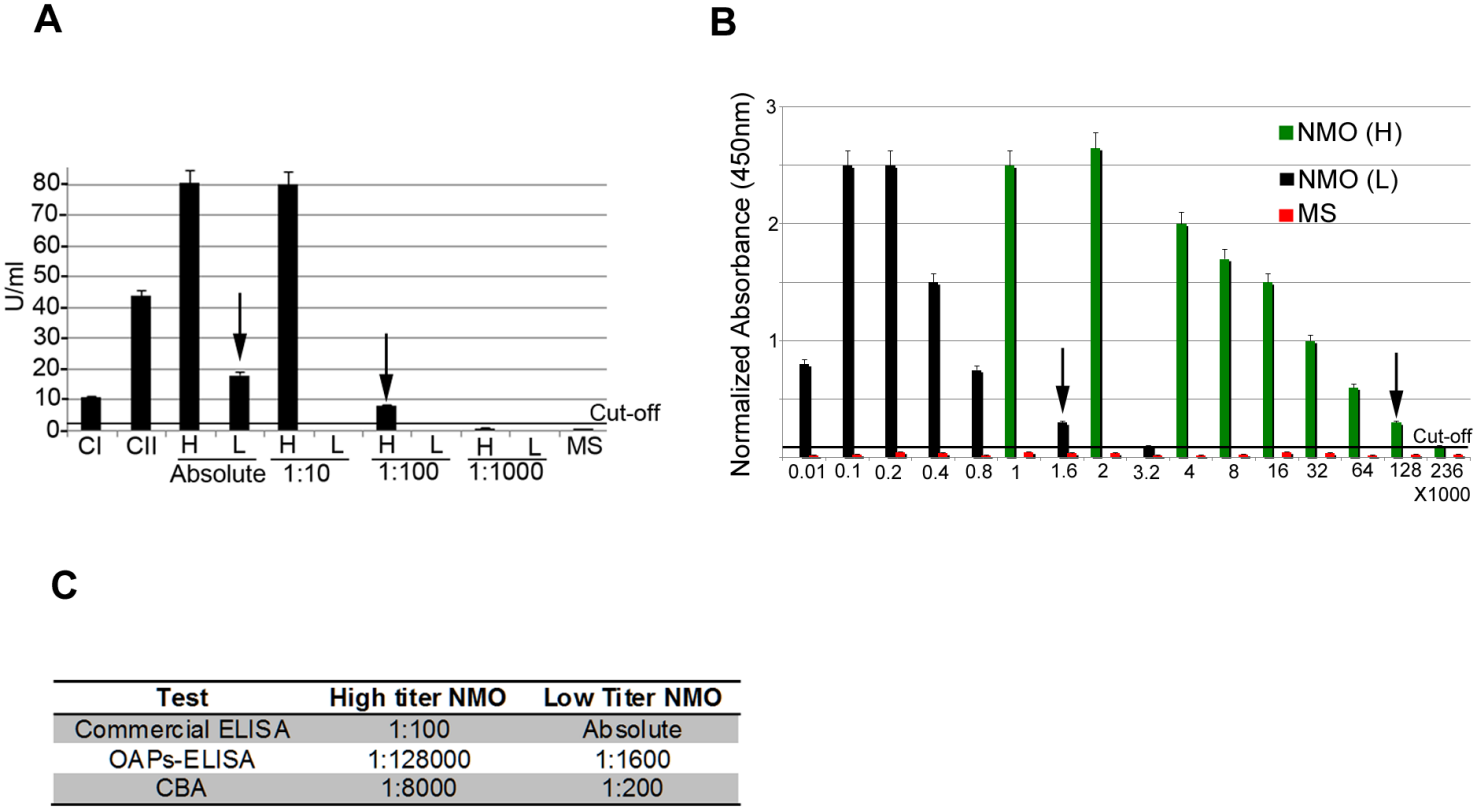
Detection limits of commercial ELISA, OAP-ELISA and CBA. (A) Positive controls (CI, CII), High- (H), Low- (L) titer NMO sera and MS sera were tested using limiting dilution analysis by commercial ELISA. (B) MS and H and L NMO sera serial dilutions tested by OAP-ELISA. Arrows highlight end-point titer. (C) Table summarizing the end-point titer obtained with commercial ELISA, OAPs-ELISA and CBA.

End-point titer of OAP-ELISA was compared to that of commercial ELISA and to our in-house CBA. Two representative High titer (H) and Low titer (L) NMO sera were tested by commercial (Fig 7A) and OAPs-ELISA (Fig 7B) by serial dilutions. For H-sera, the limiting dilutions were 1:100 and 1:128,000, for commercial and OAPs-ELISA respectively, while for L-sera they were absolute and 1:1600 respectively. For OAPs-ELISA using 1:10 L-serum the normalized absorbance was affected by high non-specific IgG binding in the negative control empty well. With CBA the NMO-IgG detection limits were 1:8,000 for the H-serum and 1:200 for the L-serum. End-point titer of OAPs-ELISA was further confirmed using NMO and control pooled sera (S2 Fig). These data demonstrate that OAPs-ELISA is at least one thousand times more sensitive than the commercial ELISA and up to 6 times more sensitive than the CBA test.

## Discussion

Since the discovery of AQP4-IgG as a diagnostic and pathological hallmark for NMO, different approaches have been proposed for its detection in patients’ serum. The IPND has underlined the importance of progressively improving the sensitivity for AQP4 IgG detection since the sero negativity for AQP4 IgG could in some cases be due to technical limits.

Currently, the CBA is the gold standard technique, recommended by the IPND, which however underlines that this test is still not yet widely available and it needs to be referred to a specialized laboratory [16]. Moreover, we have previously shown [2]that several technical aspects may affect its sensitivity.

Protein-based tests, such as ELISA, may potentially solve a series of CBA critical steps. However, although a few laboratories have developed ELISA tests, and a commercial kit is available, their sensitivity is too low to represent a valid alternative to CBA. Indeed, for in-house ELISA a sensitivity of 71% has been reported using recombinant rat M23 with N-terminal His-tag [25], and a sensitivity of 72% using human M23 with the coexistent presence of C- and N- terminal His tags [30]. Regarding the commercial kit, two versions of the RSR^TM^ELISA have been produced with M1-AQP4 (first version) and M23-AQP4 (second and latest version). Using the commercial kit with M1, 48.3% sensitivity has been reported by Isobe [37], 58% sensitivity has been reported by Fryer [27] and 75.8% sensitivity has been reported by Jarius [28].

Based on our previous study demonstrating that AQP4 organization into OAPs is key for AQP4-IgG binding (13), our hypothesis is that a pivotal solution to improve the ELISA approach is based on the use of integral AQP4-OAP as a molecular target. In contrast with our view however, other studies have reported that by fluorescence immunoprecipitation assay (FIPA), using M1 transfected cells with an N-terminal GFP tag, was able to detect NMO-IgG with 53.3% and 76% sensitivity [38,39].

The main obstacle to OAP use in ELISA is the technical procedure required to isolate intact OAPs recognized by AQP4-IgG. Here we faced this problem by developing a new method to isolate integral OAPs from CNS, using nSEC. We here demonstrate that when used on plastic 96-well plates these OAPs are recognized by AQP4-IgG with 91% sensitivity and 99% specificity. Based on our results we can presume that the reported low sensitivity of the commercial ELISA may be due to the low level of functional OAPs in this kit. In these tests, the use of an N-terminal His tag on M1 and M23 expressed in insect cells [25,30] could have compromised OAP assembly as already reported [2]. We cannot rule out, however, that the differences observed might be due to differences related to technical conditions such as the buffers, the amount of proteins in the wells, or the detection system.

The important aspects that need to be evaluated to develop an efficient AQP4-ELISA are the OAP composition, the expressing system and the technical approach used for AQP4 purification. Here we have developed an OAPs-ELISA based on native rat brain OAPs, which potentially reproduces the complexity of a natural NMO-IgG target, and biochemical purification with few steps. Indeed, the single step nSEC resulted in rapid and efficient OAP isolation with a high degree of purification and with NMO-IgG epitope preservation.

The approach here reported represents, nowadays, the key solution for a high end-point titer of the ELISA method. Furthermore, subsequent biochemical analysis of the OAPs obtained with this nSEC protocol may provide new information on the fine OAP composition and on key factors that may result in the optimization of ELISA using heterologous expression systems such as insect cells.

The step forward of the present study resides in two important aspects. One is the possibility of easy access for a wide range of laboratories to a simple AQP4 IgG ELISA test. The CBA test is in fact not widely used since it requires specialized laboratories and particular technical expertise for the analysis of the results obtained. The other important aspect here shown is the sensitivity, even higher than the current gold standard CBA test.

In conclusion, this study represents the first attempt at developing an OAPs-based ELISA to detect AQP4-IgG in patient sera representing a valid alternative to the CBA. We believe that our findings lay the foundations to develop a new generation of AQP4-IgG detection methods in which an easier and more accessible ELISA test will replace the more difficult CBA test as the gold standard, therefore facilitating earlier and more accurate diagnosis.

## Supporting Information

S1 Fig Four low absorbance NMO sera were retested confirming their positivity. (TIF)

S2 Fig End-point titer of OAPs-ELISA using pooled sera. Low (L) and high (H) titer pooled sera were tested between 1:1000 to 1:128.000. Note the high end-point titer of both NMO categories.
(TIF)

S1 Table Comparison between OAPs-ELISA and Commercial CBA sensitivity and specificity. (DOCX)
