# Supplementary figures and images for: Development of an Aquaporin-4 Orthogonal Array of Particle-Based ELISA for Neuromyelitis Optica Autoantibodies Detection
Source: PLoS One. 2015 Nov 24;10(11):e0143679. doi: 10.1371/journal.pone.0143679 (PMC4658006; doi:10.1371/journal.pone.0143679)

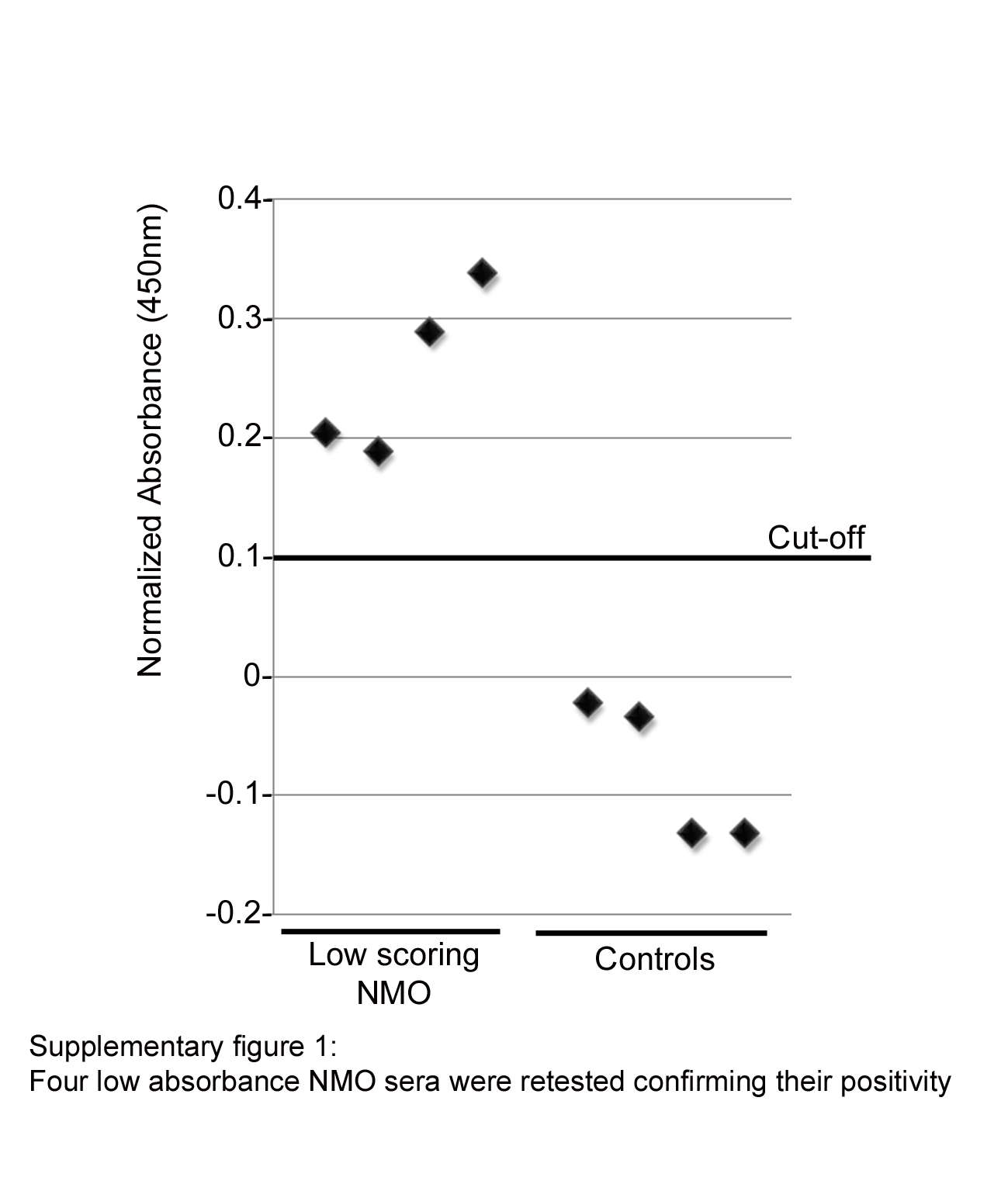

Supplement: S1 Fig — (TIF) [file pone.0143679.s001.tif]

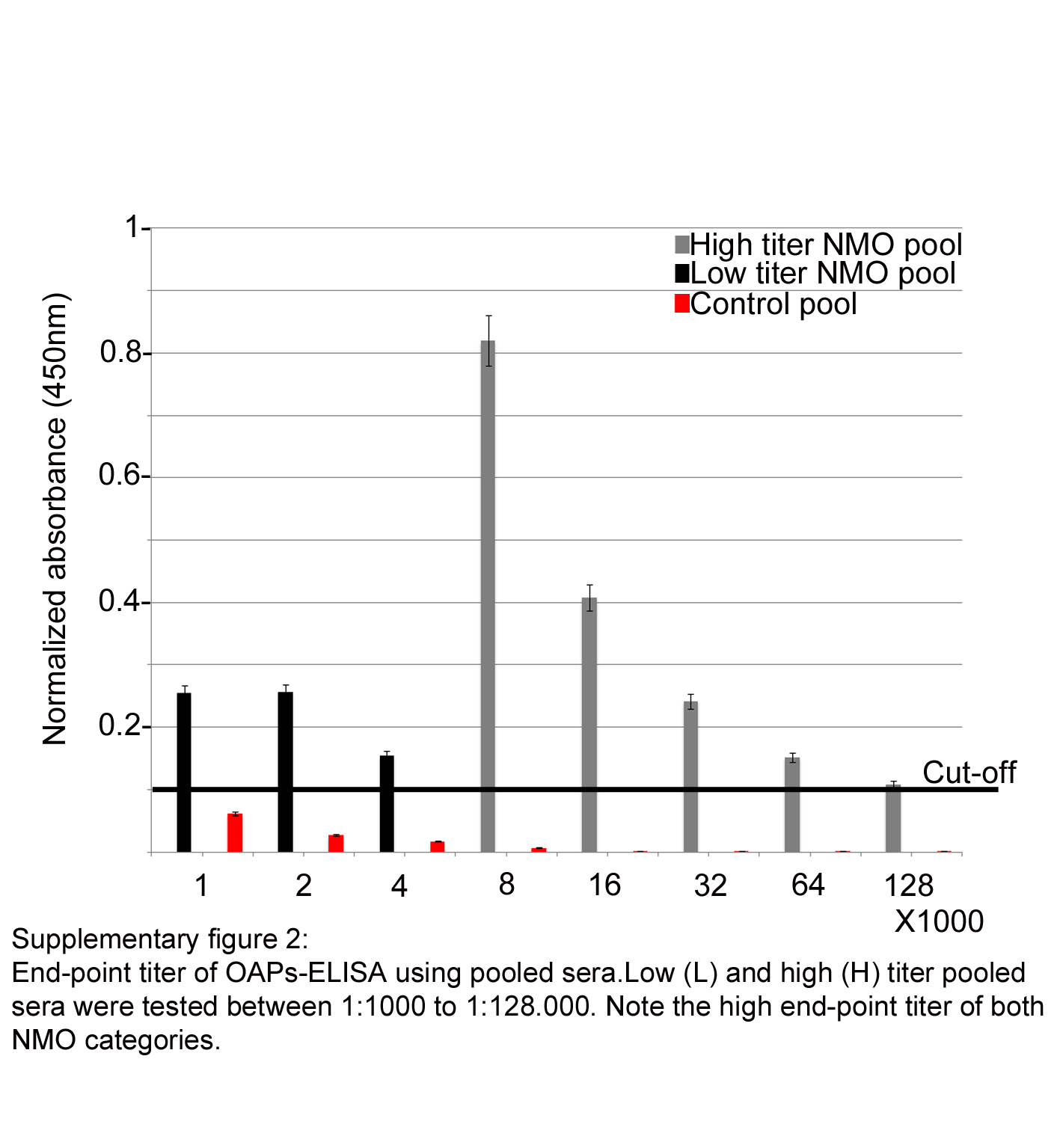

Supplement: S2 Fig — Low (L) and high (H) titer pooled sera were tested between 1:1000 to 1:128.000. Note the high end-point titer of both NMO categories. (TIF) [file pone.0143679.s002.tif]
