# Supplementary material for: Development of an Aquaporin-4 Orthogonal Array of Particle-Based ELISA for Neuromyelitis Optica Autoantibodies Detection
Source: PLoS One. 2015 Nov 24;10(11):e0143679. doi: 10.1371/journal.pone.0143679 (PMC4658006; doi:10.1371/journal.pone.0143679)
Supplement: S1 Table — (DOCX) [file pone.0143679.s003.docx]

| Patients (45) | OPAs-ELISA positive | Commercial CBA |
| --- | --- | --- |
| NMO (25) | 23 | 19 |
| Controls (20) | 1 | 0 |
| Sensitivity | 92% | 76% |
| Specificity | 95% | 100% |

**Supplementary table**

Comparison between OAPs-ELISA and Commercial CBA sensitivity and specificity.
